# Cr-Al Spinel phase formation in alumina dispersed 316 L stainless steel processed by spark plasma sintering

**DOI:** 10.1038/s41598-025-87223-0

**Published:** 2025-03-01

**Authors:** Zsolt Czigány, Haroune Rachid Ben Zine, Katalin Balázsi, Csaba Balázsi

**Affiliations:** 1 Institute of Technical Physics and Materials Science, HUN-REN Center for Energy Research, Konkoly-Thege M. St. 29-33, Budapest, 1121 Hungary; 2 Faculty of Sciences and Technology, Mohamed Khider University, BP 145 RP, Biskra, 07000 Algeria

**Keywords:** Cr-Al Spinel, Oxide dispersion strengthened steel (ODS), Spark plasma sintering (SPS), Mechanical alloying (MA), Transmission Electron Microscopy (TEM), Structural materials, Techniques and instrumentation

## Abstract

Phase transformation of oxide phase in oxide dispersion strengthened (ODS) 316 L stainless steel alloys was observed during spark plasma sintering (SPS).The composites were prepared with two different compositions of 0.33 wt% Al_2_O_3_ and 1wt% Al_2_O_3_. The alumina particles were located at grain boundaries mixed with micrometer sized steel debris from milling after attrition milling. The alumina particles transformed to a Cr-Al spinel phase dominantly with Cr rich composition surrounded by an amorphous silica phase during SPS process in both sintered composites. Both Cr component of Cr-Al spinel phase and Si in silica could diffuse from the 316 L steel during the spark plasma sintering process. The lattice parameter of the spinel phase is 8.36Å independent of the local cation composition variation. The lattice parameter of the spinel phase is relatively large among synthetic Cr-Al spinels which implies that octahedral sites of spinel structure are mainly occupied by Cr^3+^ cations replacing a portion of Al. The finding that the transformation occurs in presence of amorphous silica is consistent with literature describing both geological occurrence of chromite and phases with spinel structure in annealed glass composites in the presence of silica phase. The phase transition may be also promoted by local temperature increase at the grain boundaries of steel during the spark plasma sintering.

## Introduction

Spinel structures are classified into 3 groups of oxyspinel, thiospinel and selenospinel according to Bosi et al.^1^. These groups are represented by the formula AB_2_ × _4_ where A and B are cations, where B is the constituent with valence 3 + and X represent the anions. In some cases, the cations in T (tetrahedral) and M (octahedral) positions are written together between brackets. The formula A^2+^B_2_^3+^O_4_ represent the oxyspinel group, where generally, the A and B positions are occupied by 2 + and 3 + valence cations, respectively. Chemically, the spinel structure can accommodate different atoms with different charges, sizes and also vacancies^1^. Also it is known that spinel structures do not always have the above described configuration of the A cations in the tetrahedral positions and the B cations in the octahedral positions, therefore, a spinel structure can be represented by the following general formula ^IV^(A_1 − i_ B_i_)^VI^(A_i_B_2−i_)O_4_ where “i” is the inversion parameter (normal spinel i = 0, inverse spinel i = 1)^2^, IV and VI denotes the 4 and 6 fold coordination of cations in tetrahedral and octahedral sites, respectively.

Chromite is a relevant spinel related to the present study. The ideal formula of pure chromite is Fe^2+^Cr_2_^3+^O_4_ but this form is rare in nature. Common substitutions are Mg for Fe and Al for Cr, therefore, the general formula chromite is (Fe^2+^,Mg^2+^)(Cr^3+^,Fe^3+^,Al^3+^)_2_O_4_. In geological terms chromitite is a rock consisting predominantly of chromite. (Chromitite is not to be confused with the non-related compound of chromatite (CaCr^6+^O_4_) which has a tetragonal structure and space group of *I*4_1_/*amd*).

In nature, chromite forms in mafic and ultramafic (low silica content) igneous rocks and is also found in metamorphic rocks such as serpentinites^3^. Its occurrence is associated with layered mafic intrusions, where it crystallizes from a magma or molten lava^3^. The geological study of chromite containing layers provides valuable insights into the Earth’s mantle processes and the differentiation of the Earth’s crust. Chromitite is an important mineral source of chromium^4^. Ferrochrome (an alloy of chromium and iron), extracted from chromitite is a key ingredient in stainless steel production. Spinels are also identified as part of the mineralogical assemblages in silicate inclusions of iron meteorites [Bunch]. These spinels typically include chromite (FeCr₂O₄) and are sometimes enriched with titanium, aluminium, and other trace elements. The inclusions themselves are similar in silicate mineral composition and mineral assemblage to terrestrial ultramafic rocks.

Several efforts can be found in the literature for synthesis of compounds with spinel structure from mixtures of binary oxides of the components. A condensed-state reaction between A1_2_O_3_ and MgO crystals conducted in air at 1560 °C was performed by Rossi^5^ to produce MgAl_2_O_4_ spinel. For the same purpose Navias applied MgO vaporization by heating in the range of 1500 °C to 1900 °C in a hydrogen atmosphere and the vapor products diffused into Al_2_O_3_^6^. In similar experiments Carter investigated the counterdiffusion of the Mg^2+^, Fe^3+^, and Al^3+^ ions through the relatively rigid oxygen lattice of the spinel structure in the solid-state reactions during formation of MgAl_2_O_4_ and MgFe_2_O_4_ at 1380 °C^7^. The possibility of formation of CrAl_2_O_4_ was shown from the equimolar mixture of co-precipitated Al_2_O_3_ and Cr_2_O_3_ oxides under a reductive environment at 1150 °C^8^. The cell parameter of the formed CrAl_2_O_4_ spinel was 8.22(3) Å. A FeCr_2_O_4_ spinel phase in Fe_2_O_3_-Cr_2_O_3_ mixtures can develop at 1380 °C in air and 1445 °C in oxygen^9^. The spinel phase is present at 1500 C° in air if the critical amount of 57% iron oxide is exceeded, however, the value of this critical amount decreases with addition of alumina^9^. A recent experiment demonstrated nucleation of spinel phase at an interface between two solids by Jáger et al.^10^ who annealed crystalline ZnO and amorphous alumina bilayers at 700 C. The nucleation and growth of the ZnAl_2_O_4_ spinel phase occurred in the direction of the amorphous alumina layer leaving Kirkendall voids at the interface. This is an indication that the process is controlled by the diffusion of oxygen and zinc ions of ZnO. Recent experiments report formation of spinel phase in annealed glass samples for their magnetic^11^ and catalytic^12^ properties. In both cases the mixture of precursors was produced by sol-gel method and contained precursors of silica. Gharagozlou report formation of cochromite (CoFe_2_O_4_)^11^ by annealing mixture of tetrakis(2-hydroxyethyl) orthosilicate as a water-soluble precursor of silica and Fe and Co metallic nitrates at temperatures varying from 400 to 1000 °C to study structural and magnetic properties of the formed nanocomposites. Khan et al. produced hercynite (FeAl_2_O_4_)^12^ by annealing glass samples with composition xFe_2_O_3_, yAl_2_O_3_ and (100-x-y)SiO_2_ at 1000 °C for 100 min and observed its photoactivity. The lowest process temperature applied for spinel phase formation was achieved by wet chemical process^13^ and hydrothermal and thermo-vapour conditions^14^ for franklinite and gahnite, respectively. Nanocomposites of franklinite (ZnFe_2_O_4_) doped ZnO were synthesized by wet chemical (co-precipitation) technique followed by heat treatment for two hours at 300 °C using metal chlorides and metal oxides as precursors^13^. Gahnite (ZnAl_2_O_4_) was obtained by treatment of a mixture of zinc oxide and oxide or hydroxide of aluminium at 180–400 °C in water or water vapour under pressure of 1–26 MPa^14^.

In our recent research we have investigated oxide dispersion strengthened steel formation by use of novel sintering techniques like spark plasma sintering (SPS) method^15^ when a sequence of electric pulses are applied at high temperature and pressure. It provides a means of precious modification of the kinetics of densification, reactions between oxide and matrix phases involving the possibility of forming complex oxides, including spinel phases. Spark plasma sintering (SPS) can accelerate the densification of materials that are hard-to-sinter and allow to control the atomic diffusion behaviour, phase stability surface conditions and crystal growth behaviour^16^. SPS or plasma activated sintering (PAS) and field assisted sintering (FAST), makes it possible to prepare fully densified composites at comparatively lower temperature with substantial short holding^17^. SPS has been used to consolidate different oxides, nitrides, carbides and a wide range of ceramic based composites^18^. The SPS technique is very similar to the standard hot pressing, where the starting powders are placed in a graphite die and a uni-axial pressure is applied during the sintering. Nevertheless, instead of applying an external heating source, a current, which is usually a few thousands of Ampers can pass either through the graphite die or the pressed powder sample or alternatively through both^19^. The resistance heating occurs when the die acts as a heating source and the conduction is performed along the graphite pressing tool. In the other hand when the conduction path involves the powder sample, breakdown, arcing, spark or plasma may be generated among powder particles that produce a fast densification process^20^. Therefore, the SPS process may induce densification of powder samples without substantial grain growth which can be accomplished within few minutes^15,21,22^.

In this paper we investigate the formation of the spinel phase with Cr_2_AlO_4_ composition during spark plasma sintering of oxide dispersion strengthened (ODS) 316 L stainless steel alloys with two different compositions of 0.33 wt% and 1wt% Al_2_O_3_. We intend to discuss the complex transformation mechanism in the context of other spinel and chromite phases and interpret the observed lattice parameter of 8.36Å in terms of site occupancy.

## Experimental

The commercial austenitic 316 L stainless steel (Höganäs, 316 L) with the composition of 16.8Cr-12Ni-2.5Mo-1.5Mn-0.6Si and ~ 70 μm average particles size has been milled separately (reference sample) and together with the ultra-fine Al_2_O_3_ powders (Almatis GmbH) with average grain size of ~ 200 nm^23^. The attritor milling (Union Process, type 01-HD/ HDDM) has been used for dispersion of the Al_2_O_3_ particles in the steel matrix and for a simultaneous size reduction of 316 L grains at 600 rpm in ethanol for 5 h^23^. Stainless-steel tank, agitator, and grinding media of 3 mm diameter have been used for milling^15,23^. Spark plasma sintering (SPS, Sinter-SPS-7.40MK-VII) has been used for sintering the milled powders at 900 °C under 50 MPa mechanical pressure for 5 min in vacuum (6 Pa)^15^. Sintered solid disks with ~ 100 mm diameter and ~ 9 mm thickness have been obtained. Structural, morphological and compositional results of the base powder, milled, and sintered samples measured by Scanning Electron Microscopy equipped with Energy Dispersive Spectroscopy (EDS) are published in^23^. The microstructure and composition of the sintered samples were studied by transmission electron microscopy (TEM) including high resolution TEM (HRTEM) and selected area electron diffraction (SAED) using a Cs corrected Themis TEM (Thermo Fisher Scientific, Waltham, MA, USA). Analysis was performed at 200 kV accelerating voltage with point resolution of 0.08 nm. EDS elemental maps were recorded in scanning transmission electron microscopy (STEM) mode. TEM specimens were made by Ar ion milling at incidence angle of 4° at 10 kV using Technoorg Linda equipment. The thinning procedure was finished at low-energy of 3 kV in order to minimize the possible ion beam damage^24^. SAED patterns were recorded by the standard method described in^25^ and evaluated by ProcessDiffraction Version 8.7.1 software (https://public.ek-cer.hu/~labar/ProcDif.htm)^26^.

## Results

The distribution of the ultrafine Al_2_O_3_ particles embedded in the surfaces of the milled 316 L SS grains were investigated by SEM and EDS^23^. The SEM results showed that the ultrafine (< 200 nm) alumina particles are homogenously distributed and embedded on the surface of the 316 L SS grains^23^. In the case of the 316 L/ 1 wt% Al_2_O_3_ milled powder it was observed that the steel grain surfaces were totally covered by the embedded ultrafine alumina particles resulting in the formation of a thin layer of alumina^23^. In the case of the 316 L/ 0.33 wt% Al_2_O_3_ milled powder a homogenous distribution of the ultrafine alumina particles have been observed on the surface of the steel grains^23^.

**Fig. 1:**
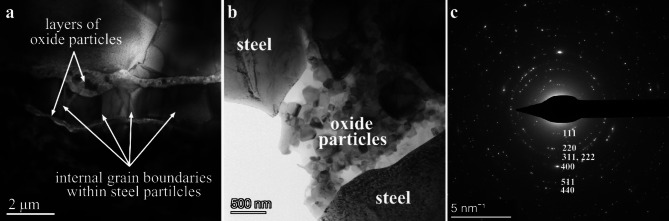
(**a**) overview and (**b**) medium magnification TEM images of the sintered composite 316 L/0.33 wt% Al_2_O_3_. (**c**) The SAED pattern of the oxide particles is shown. The diameter of selected area was ~ 600 nm.

TEM investigation of the sintered 316 L/ 0.33wt% Al_2_O_3_ grain boundaries (Fig. 1) revealed two different arrangements of the oxide particles in the grain boundaries: continuous thin layers (200–500 nm wide) along the grain boundaries of steel particles (Fig. 1a) which can form agglomerates at some locations mixed with fractions of the 316 L debris (Fig. 1b) and grain boundaries without oxide particles (Fig. 1a). The absence of oxide particles in these internal stainless steel grain boundaries is related to the intact large 316 L grains that preserved their dimension even after intense milling. Each steel powder grain contains smaller steel grains with several internal grain boundaries, albeit the oxide particles were embedded only to the outer surface of these grains. The selected area electron diffraction (SAED) pattern of 316 L/ 0.33wt% Al_2_O_3_ composite (Fig. 1c) revealed a spinel phase (space group: F d -3 m) with lattice parameter of 8.36Å which was confirmed by Fast Fourier transform (FFT) of the high-resolution (HRTEM) images. Since reflections of residual Al_2_O_3_ phase was not observed (neither in SAED nor in FFT of the high-resolution images), the Al_2_O_3_ oxide has completely transformed to Cr-Al spinel phase in 316 L/ 0.33wt% composite.

**Fig. 2:**
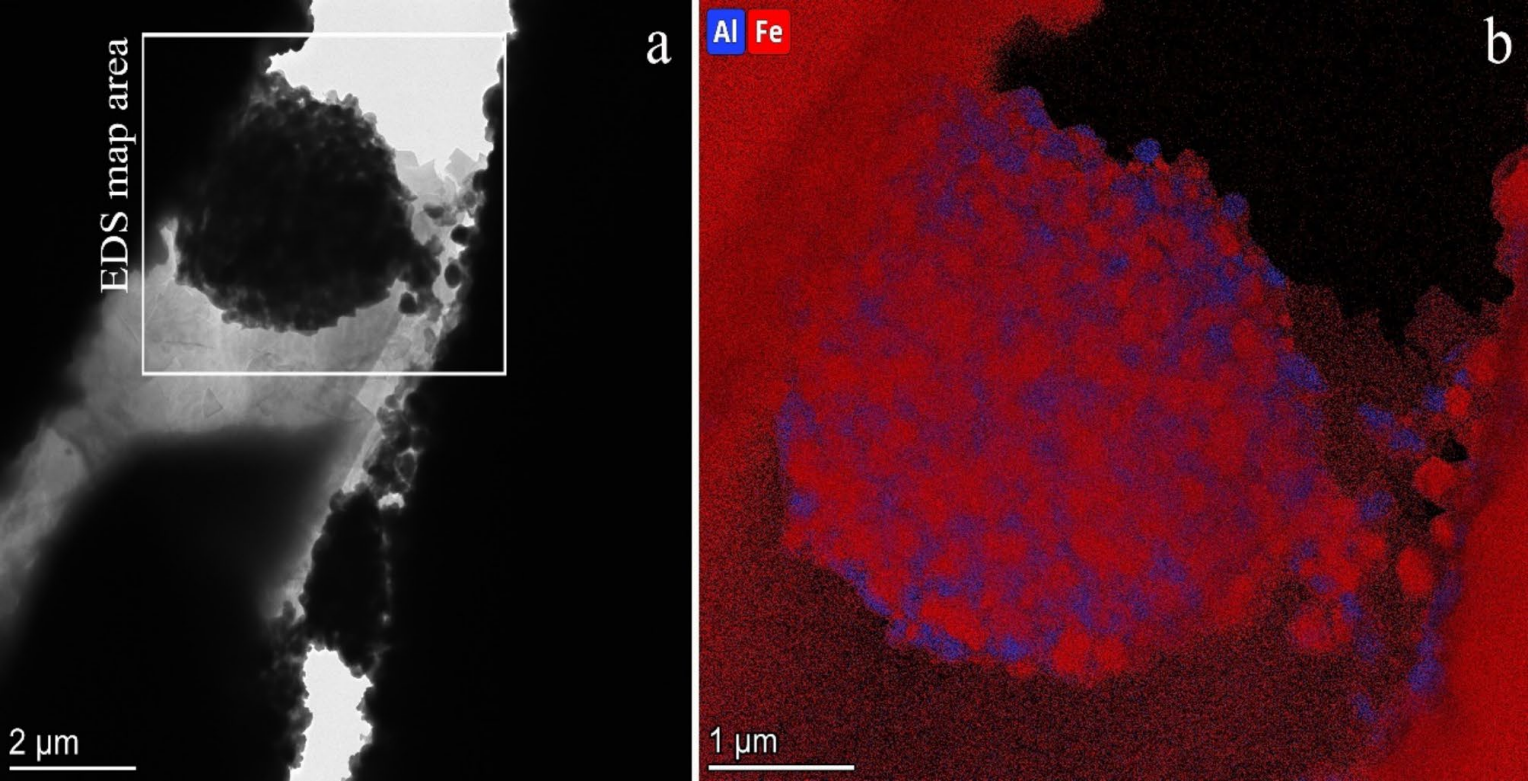
(**a**) Overview TEM image of the sintered composite 316 L/1 wt% Al_2_O_3_. (**b**) Combined Al-Fe elemental map of the spherical aggregate highlighted in panel (**a**). The intensity in the elemental map is proportional with atomic concentration.

**Fig. 3:**
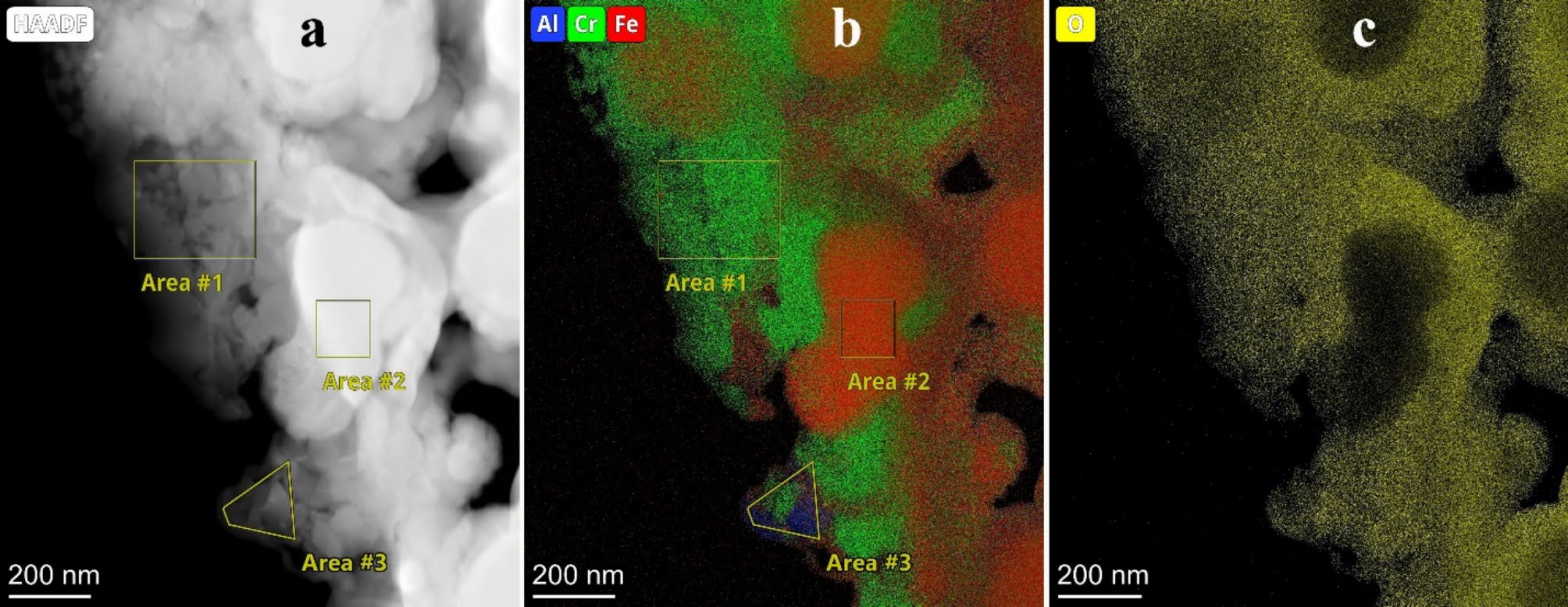
(**a**) HAADF image and (**b**-**c**) the related elemental maps of the sintered 316 L/ 1wt% Al_2_O_3_ grain boundaries. The intensity in the elemental map is proportional with atomic concentration.

In the case of the 316 L/ 1wt% Al_2_O_3_ composite (Fig. 2), similar results to the 316 L/ 0.33wt% Al_2_O_3_ were observed. Obviously, higher amount of oxide particles between the steel grains resulted in larger, some micrometre sized agglomerates (Fig. 2a). The Al-Fe elemental map of an aggregate (Fig. 2b) illustrates the arrangement of Al containing oxide particles and steel debris. Similarly, a mixture of oxide particles and steel debris is shown for 316 L/ 0.33wt% Al_2_O_3_ composite in Fig. 1b. Based on SAED investigation of the large agglomerate in Fig. 2, the same spinel structure was observed for the oxide phase in 316 L/ 1wt% Al_2_O_3_ composite like in 316 L/ 0.33wt% Al_2_O_3_ composite with the same lattice parameter. At some locations high Al concentration is detected in the oxide phase by compositional analysis (see Area3 in Fig. 3b) which are indications of residual Al_2_O_3_. Note, that the amount of the residual Al_2_O_3_ is very small and the oxide phase is almost completely transformed to Cr-Al spinel in 316 L/ 1wt% Al_2_O_3_ composite as well.

**Fig. 4:**
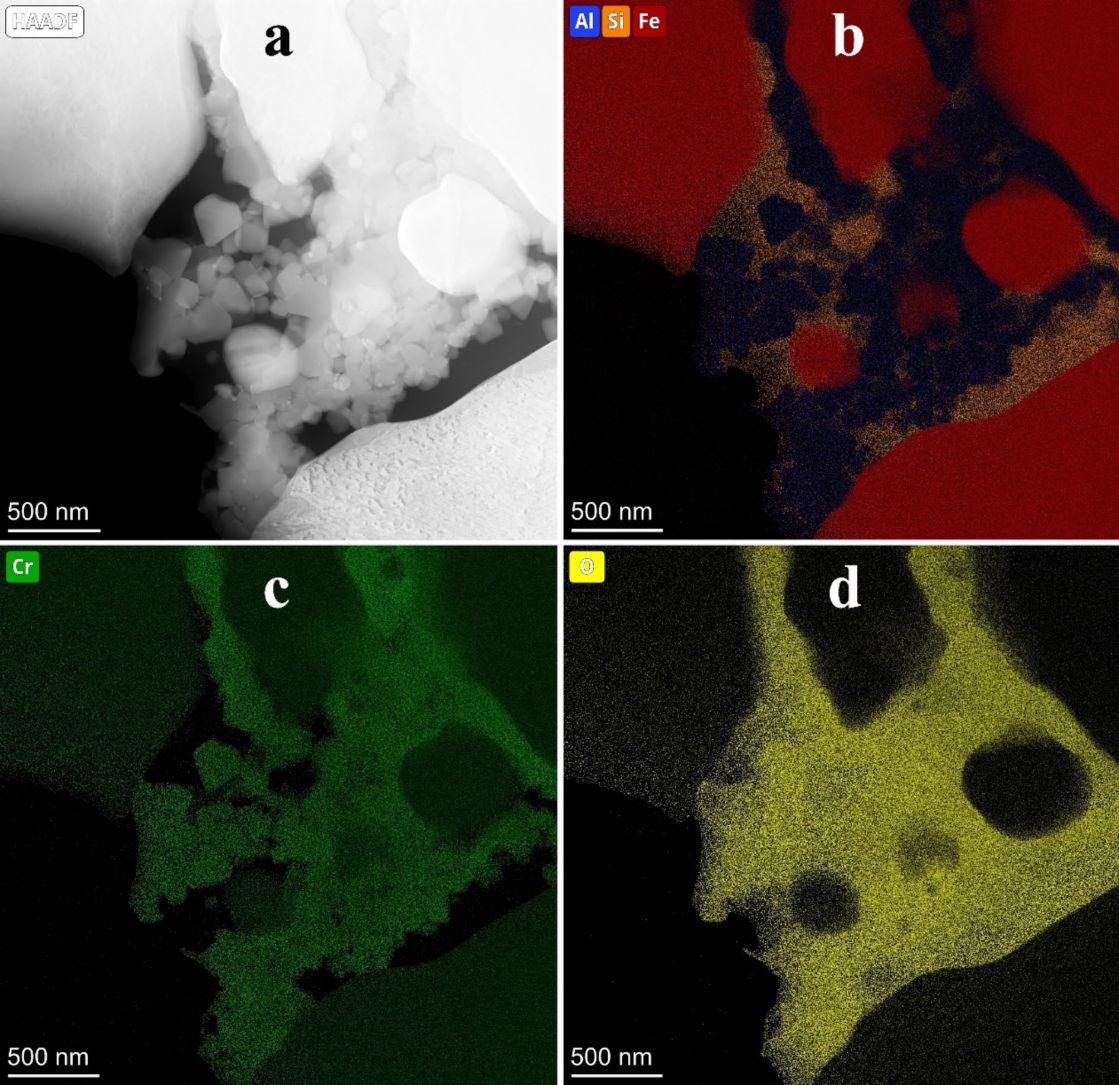
(**a**) HAADF image and the (**b**-**d**) related elemental maps of the sintered 316 L/ 0.33 wt% Al_2_O_3_. High oxygen content up to 60 at% was measured among the steel particles. The combined AlSiFe map illustrates the complementary nature of Al (Cr-Al spinel phase) and Si (silica) between steel grains. The spinel phase particles are also rich Cr. (Note that Cr content in the Cr-Al spinel particles is higher than ~ 16at% Cr content of 316 L steel!). The intensity in the elemental maps is proportional with atomic concentration.

Figure 4 shows the STEM EDS analysis of an agglomerate of oxide particles between steel grains in sintered 316 L/ 0.33 wt% Al_2_O_3_ composite The high-angle annular dark-field (HAADF) image (Fig. 4a) and the corresponding elemental maps (Fig. 4b-d) show that the oxide agglomerate consists of nano- and ultrafine grains rich in Al, Cr and O surrounded by an amorphous silicon oxide phase with an approximate composition of SiO_2_. The silica content within the intergrain oxide region is ~ 25vol%. The EDS results show that the Cr content is about 2 times higher than Al. Also, high oxygen content up to 60 at% has been measured which is consistent with oxygen content of spinel and silica phases.

Figure 3 shows the STEM EDS analysis of a thin section of an agglomerate of oxide particles between steel grains in sintered 316 L/ 1 wt% Al_2_O_3_ composite. The high-angle annular dark-field (HAADF) image (Fig. 3a) and the corresponding elemental maps (Fig. 3b-c) show that the oxide agglomerate consists of nano- and ultrafine grains rich in Cr, Al and O. Among the three regions of interests, Area1 and Area3 highlight two regions of oxide particles with very different composition (Table 1). Area2 is a steel debris particle. Area1 is a high Cr content region with small amounts of Al and Fe has a spinel structure by SAED. The Cr rich spinel structure is quite similar to that observed in 316 L/ 0.33 wt% Al_2_O_3_ composite and can be considered as typical for 316 L/ 1 wt% Al_2_O_3_ composite as well. In Area3 a significant compositional variation was observed with high Al content combined with smaller amounts of Cr and Fe. Such area, where the transformation to Cr-Al spinel phase is not complete, is rather exceptional in the composite. In both areas high oxygen content up to 60 at% has been measured (consistent with oxygen content of spinel and silica phases). It is also a remarkable difference that the silica content in Area1 is ~ 25vol% like in 316 L/ 0.33 wt% Al_2_O_3_ composite, while in Area3 the silica content is negligible. In the SAED pattern the same spinel phase was observed both in Area1 and Area3 with lattice parameter of 8.36Å. Based on FFT of high resolution TEM images, variation by location of lattice parameter of the spinel phase was not observed.

**Table 1:** Comparison of the elemental composition of the selected areas 1 and 3 in Fig. 3.

			Area1	Area3
**Z**	Element	X-ray line	Atomic fraction and error (%)	Atomic fraction and error (%)
**8**	**O**	K	**59.67** ± 2.6	**59.59** ± 2.65
**13**	**Al**	K	**2.2** ± 0.44	**20.27** ± 3.22
**14**	**Si**	K	**1.83** ± 0.36	**0.03** ± 0.02
**24**	**Cr**	K	**31.74** ± 2.91	**10.26** ± 1.3
**26**	**Fe**	K	**4.03** ± 0.54	**8.3** ± 1.08
**28**	**Ni**	K	**0.38** ± 0.06	**1.27** ± 0.19
**42**	**Mo**	K	**0.15** ± 0.03	**0.29** ± 0.07

## Discussion

During our spark plasma sintering of oxide dispersion strengthened (ODS) 316 L stainless steel alloys with two different compositions of 0.33 wt% and 1wt% Al_2_O_3_ we observed the formation of the spinel phase with Cr_2_AlO_4_ composition with some composition variation in 316 L/ 1wt% Al_2_O_3_ composite. The transformation of Al_2_O_3_ to spinel phase was complete in case of sintered 316 L/ 0.33wt% Al_2_O_3_ and no residual corundum Al_2_O_3_ component was detected in SAED patterns of HR images. Only a negligible amount of residual alumina was detected in sintered 316 L/ 1wt% Al_2_O_3_ composite. The lattice parameter of the spinel phase was 8.36Å, independent of the composition. The oxide structure is analogous with the spinel CrAl_2_O_4_ which has a calculated lattice parameter of 8.106 Å (ICSD-122663 card;^8^) and 8.22 Å was measured by Shtyka et al.^8^. However, according to our EDS measurements in our sintered 316 L/ Al_2_O_3_ composites there is typically ~ 2 times more Cr than Al and also a significant cation composition variation was observed in 316 L/ 1wt% Al_2_O_3_ composite (Table 1). According to Shtyka, Cr and Al bearing spinels can have a large compositional variation related to either high-Al or high-Cr variants^8^.

In geology, chromite and spinels generally form in mafic and ultramafic igneous rocks^3^ within a temperature range of 900 °C to 1200 °C. The pressure conditions can vary significantly depending on the specific geological setting, ranging from low-pressure environments in the Earth’s crust to high-pressure conditions in the Earth’s mantle. For chromite formation specifically, pressures can range from a few 100 MPa to higher pressures of over 1GPa^27^. In addition to pressure and temperature oxygen fugacity is a decisive parameter for chromite formation^27^. Aspects of spinel occurrence in mineralogical assemblages in silicate inclusions of iron meteorites are discussed in Bunch et al.^28^.

On the other side, it is also important to note that too high pressure and/or temperature can be detrimental for the stability of spinel structure. Kyono et al.^29^ proved that the crystal structure of chromite is transformed from a cubic to a tetragonal structure between 11.8 and 12.6 GPa. Formation of other chromite polymorphs, like chenmingite and xieite from chromite precursor require 16-18GPa pressure^30^. Chenmingite can form below ~ 1350 °C but xiete requires higher temperature. Such high temperature and high pressure conditions may be induced impact shock of meteorites. Similarly, high-pressure high-temperature transitions in MgCr_2_O_4_ was studied in^31^ up to 1600 °C and 28GPa. Also important to note the influence of the chemical properties of the ambient: high temperature can also induce reduction of chromite to ferrochrome in reductive ambient as 900–1200 °C treatment is applied for industrial ferrochrome production from chromite ore^32^.

Laboratory efforts to synthesize compounds with spinel phase from mixtures of binary oxides of the components generally reported higher temperatures compared to geological conditions and generally performed at atmospheric pressure. Note also that the some hours’ time scale of laboratory experiments is orders of magnitude shorter than that of geological processes. For condensed-state reaction between Al_2_O_3_ and MgO crystals in air Rossi^5^ reported 1560 °C transformation temperature. Gas phase reaction of the same materials was performed in the range of 1500 °C to 1900 °C in a hydrogen atmosphere^6^. FeCr_2_O_4_ spinel phase in Fe_2_O_3_-Cr_2_O_3_ mixtures can develop at 1380 °C in air and 1445 °C on O_2_^9^. However, it is important to emphasize the complexity of the processes. In these transformations ion substitution depends very much on the chemical ambient, i.e. reductive or oxidative conditions^6^. It is an interesting observation in the experiment of Muan et al., that the spinel phase is present at 1500 °C in air if the critical amount of 57% iron oxide is exceeded, however, the value of this critical amount decreases with addition of alumina^9^, suggesting a catalytic-like effect of alumina.

Cr and Al bearing spinels are characterized by the general formula (Cr, Fe)(Cr, Al, Fe)_2_O_4_ and are part of a broader family of spinel minerals. Generally Fe and Cr can occupy both tetrahedral (Fe^2+^, Cr^2+^) and octahedral (Fe^2+^, Cr^2+^) sites in spinel structure, however, Al can primarily occupy octahedral sites as Al^3+^. In chromite, Al^3+^ can substitute for Cr^3+^ in the octahedral position. The effort of producing CrAl_2_O_4_ by heat treatment of Al_2_O_3_ and Cr_2_O_3_ oxides at 1150 °C^8^ showed that the crystallization of atomically mixed amorphous Al oxide and Cr oxide starts with the formation of α-Cr_2_O_3_ and α-Al_2_O_3_, followed by formation of α-(Al, Cr)_2_O_3_ at 800 °C. It was proposed that at temperatures above 1150 °C, the α-Cr_2_O_3_ undergoes reduction to CrO and its Cr^2+^ ions further diffuse into the α-Al_2_O_3_ matrix, forming CrAl_2_O_4_. This mechanism of formation of spinel compounds was proposed by many researchers, particularly Rossi^5^, Carter^7^, and Navias^6^, who studied formation of magnesium aluminate spinel. Based on their results they claim that the reaction proceeds through interdiffusion of Al^3+^ and Mg^2+^ ions through the oxygen array.

In stainless steel many authors report segregation of Cr on the surface of the steel grains due to ball milling^33^. Segregation can be enhanced by high temperature, e.g. segregation of Mo, Cr, Si was observed in 316 L specimens fabricated by selective laser melting^34^. In general, the segregated Cr and its oxidation provide the corrosion resistance of stainless steels. Moreover, Yu^35^ reported detection of minor amount of CoCr_2_O_4_ spinel phase during high-temperature oxidation of and ODS steel strengthened by dispersion of Co-based oxide of Co-20Cr-(5, 10) Al-2.4Hf-1.5Y_2_O_3_(wt%) at 900 °C and 1000 °C in air. They observed that at 5wt% alumina content duplex scale of CoCr_2_O_4_/Cr_2_O_3_ developed. Liu et al. found that aluminium plays the main role in improving the oxidation resistance in ODS alloys containing Cr ≥ 14wt% by forming Al_2_O_3_ scale, also Cr_2_O_3_ transforms into FeCr_2_O_4_ in SUS430 where Fe tends to diffuse easily and form Fe-rich oxides^36^.

The mechanism of the Cr-Al spinel formation observed in our sintered 316 L/ 0.33wt% and 1wt% Al_2_O_3_ composites may be analogous with reactions both between oxide phases in ODS steels^35,36^ and between Al_2_O_3_ and Cr_2_O_3_ oxides^8^. Cr and Si segregate to the grain boundaries during the sintering process^34^ where Cr_2_O_3_ (or possibly CrO^8^) and silica is formed by reaction with oxygen. Presence of steel debris (1–2 micron particles resulted from milling) provide an increased surface for Cr and Si segregation. Reaction of oxide of surface segregated Cr and the dispersed α-Al_2_O_3_ nanoparticles can take place during SPS process performed at 900 °C sintering temperature. It is important to note that the local temperature between the grains can be higher than the nominal process temperature during current pulses in SPS process due to different resistance of the intergrain regions compared to that of steel grains^20^. In addition to grain surfaces, at current transit point between the grains hot spots can develop where the local temperature can be higher than the nominal process temperature^20^. It is quite difficult to estimate the extent of the local temperature increase. Laporte assumes presence of melt phase in these locations and development of so called “melt pockets”^37^. As described in^37^ the interfacial energy anisotropy and melt connectivity at low melt fraction in ideal case may be assumed that the melt configuration is the same along each edge of the grains. Therefore the melt distribution is homogeneous at the grain scale and may be described as a regular network of isolated pockets or interconnected channels^37^.

**Table 2:** Lattice parameter (Fe, Al)(Fe, Cr, Al)_2_O_4_ and related spinel phases.

ICSD code	a_0_ (Å)	Chemical formula	Reference
112,663	8.106 Å	CrAl_2_O_4_	^2^ calculated
	8.22 Å	CrAl_2_O_4_	^2^ experimental
187,922	8.154 Å	(Fe_0.859_Al_0.142_)(Fe_0.154_Al_1.893_Cr_0.009_)O_4_	^2^
187,923	8.326 Å	(Fe_0.988_Al_0.012_)(Fe_0.043_Al_0.475_Cr_1.483_)O_4_	^2^
187,924	8.336 Å	(Fe_0.976_Al_0.023_)(Fe_0.023_Al_0.329_Cr_1.647_)O_4_	^2^
187,925	8.355 Å	(Fe_0.986_Al_0.013_)(Fe_0.0125_Al_0.175_Cr_1.811_)O_4_	^2^
187,926	8.367 Å	(Fe_0.992_Al_0.008_)(Fe_0.0019_Al_0.071_Cr_1.925_)O_4_	^2^
171,121	8.3765	FeCr_2_O_4_ (chromite)	^2^
50,567	8.395 Å	Fe_3_O_4_ (magnetite)	^2^

There are many possible mechanisms/routes for the formation of spinel phases by exchanging the cations between the binary oxides^5–7,10^ to take place between Al_2_O_3_ and chromium oxides as described by^8^. An example for nucleation of ZnAl_2_O_4_ spinel at an interface between two solids is demonstrated in^10^ who annealed crystalline ZnO and amorphous alumina bilayers at 700 C. This observation supports that solid state reaction requires both high temperature to provide activation energy for diffusion processes and contact of reacting phases to maintain the supply of component ions. The contact of binary oxide particles is not always obvious^7,9^ and vapour phase transport between the particles require even higher temperatures, like 1500 °C to 1900 °C for vaporization of MgO^6^.

Therefore, it seems reasonable to consider the possibility of an alternative or additional mechanism which assumes that a liquid like phase mediates between the grains of the powder like in many geological occurrence of spinels where the chemical environment of mafic or ultramafic formations is characterized by low silica content^3^. Spinels identified in silicate inclusions of iron meteorites also have similar mineral composition like terrestrial ultramafic rocks^28^. The oxygen fugacity (fO₂) of the magmatic or metamorphic environment affects the oxidation state of chromium and iron^27^, which in turn influences the formation of chromite. Higher oxygen fugacity favours the formation of Cr³⁺ over Cr²⁺ and Fe³⁺ over Fe²⁺, affecting the mineral chemistry and the partitioning of these elements between the crystalline phase and its surrounding matrix. According to Irvine^39^ the increase of silica and alkalies in the basic liquid should cause it to become more polymerized with a lower frequency of octahedral sites. During crystallization, Cr^3+^ is preferentially expelled (into chromite) owing to its large octahedral crystal-field stabilization energy^38,39^.

This phenomenon that alumina can transform to a Cr rich spinel is not only surprising but it is also remarkable that significant transport of Cr can take place from the steel to the oxide phase at a relatively low process temperature of 900 °C. Based on the above discussion we propose two factors which can promote the cation transport. The first factor is the possibility of the formation of melt pockets. The process temperature in our SPS procedure is relatively low to expect a liquid phase of silica at 50 MPa (melting temperature is 1710 °C), however, temporal occurrence of several 100 °C temperature increase during SPS pulses compared to the process temperature in melt pockets is a reasonable assumption. In addition to increased temperature at melt pockets, another significant factor is the presence of amorphous silica. In case of 316 L steel the formation of a protective scale on steel surface is a collective formation process of silica (SiO_2_) and chromia (Cr oxide) from Cr and Si present in the steel alloy. Amorphous phase of silica is thermodynamically more stable at low and moderate temperatures compared to crystalline forms like quartz or cristobalite. The energy barrier for transition from amorphous to crystalline phases is relatively high and would require sustained elevated temperatures over extended periods. Beneficial effect of the presence of silica for spinel formation was reported not only for processes that take place on geological timescale and pressure conditions, but also for laboratory experiments preformed at temperatures ≤ 1000 °C^11,12^. It is also remarkable, and also in agreement with the above assumption, that in most regions in both composites the transformation of Al_2_O_3_ to Cr-Al spinel was complete where oxide crystallites were surrounded by silica and in only partially transferred region (Area3 in 316 L/ 1wt% Al_2_O_3_ composite) the silica content was negligible.

Based on the mentioned results of^5–8^ it can be proposed that the reaction proceeds through interdiffusion of Al and Cr ions through the oxygen array leading to the formation of spinel phase. According to^2^, substitutions of the Al^3+^ and Cr^3+^ within the octahedral (M) positions and Fe^2+^ and Cr^2+^↔Al^3+^ between the tetrahedral (T) and octahedral (M) positions are possible in spinels with chromium content. However, only low occurrence of Al^3+^ in tetrahedral positions may occur with inversion parameter of ~ 0.1 (see Table 2)^2^ which can broaden the possible distribution of cations within the spinel structure. In our case we observed a Cr rich spinel phase with lattice parameter of 8.36 Å in 316 L/ 0.33wt% Al_2_O_3_ composite, which can be described by the general formula of Cr(Cr, Al)_2_O_4_.

An intriguing point our observations was that the lattice parameter of the formed Cr-Al spinel is 8.36Å at every region, independently of the relative amount of metal atoms. This value is very close to the lattice parameter of the chromite FeCr_2_O_4_ 8.3765Å (ICSD_171121)^2^. In the same paper, series of Al, Cr and Fe containing spinels are investigated and a variation of lattice parameter from 8.1534(6) to 8.3672(1) Å was reported. Table 2 shows a list lattice parameters of spinels containing Al, Cr and Fe from the ICSD database. The lattice parameter, composition and site occupation information is based on^2^. Large lattice parameter between 8.336 and 8.367 Å are reported mainly for spinel compounds where Cr occupation of octahedral sites is high. Similar values of 8.3325–8.3765Å are reported by^40,41^ for (Mg, Fe)Cr_2_O_4_ spinels depending on Fe content where the octahedral sites are filled with Cr. The conclusion of these results can be that high Cr occupation of octahedral sites results high lattice parameter, which can be an explanation for the 8.36Å lattice parameter of Cr(Cr, Al)_2_O_4_ spinel formed in 316 L/ 0.33wt% Al_2_O_3_ composite. The same Cr rich Cr-Al spinel phase was observed in most regions of 316 L/ 1wt% Al_2_O_3_ composite as well with the same lattice parameter.

In the EDS analysis of Area1 (typical for 316 L/ 1wt% Al_2_O_3_) besides high Cr content and low Al content a small amount of Fe and Ni are also detected (both are components of 316 L steel). Fe and Ni cations can incorporate into the spinel structure^1^, Ni mainly in tetrahedral positions but Fe can occupy both tetrahedral and octahedral sites. Taking into account the 8.395 Å lattice parameter of Fe_3_O_4_ (Fe^2+^Fe^3 +^ _2_O_4_) magnetite (ICSD-50567) and the 8.3765Å lattice parameter of chromite FeCr_2_O_4_ (ICSD_171121), possible Fe incorporation has a minor effect on lattice parameter of the Cr rich spinel structure. Considering the low Al content as well, the most probable general formula of the formed spinel in 316 L/ 1wt% Al_2_O_3_ composite is (Cr, Fe, Ni)(Cr, Al)_2_O_4_.

## Conclusions

Oxide dispersion strengthened (ODS) 316 L stainless steel alloys with two different compositions of 0.33 wt% Al_2_O_3_ and 1wt% Al_2_O_3_ were prepared by attrition milling and spark plasma sintering process. The alumina particles located at grain boundaries transformed to a Cr rich Cr-Al spinel phase during SPS process in both sintered composites. The Cr-Al spinel crystallites are surrounded by an amorphous silica phase. Both Cr component of Cr-Al spinel phase and Si in silica could diffuse from the 316 L steel during the spark plasma sintering process. The lattice parameter of the Cr-Al spinel phase is 8.36Å in both composites, independent of the local cation composition variation. The transformation of Al_2_O_3_ to Cr-Al spinel was complete in 316 L/ 0.33wt% Al_2_O_3_ composite, while a negligible amount of residual Al_2_O_3_ was detected in 316 L/ 1wt% Al_2_O_3_ composite. The lattice parameter of the spinel phase is relatively large among synthetic Cr-Al spinels which implies that octahedral sites of spinel structure are mainly occupied by Cr^3+^ cations. In comparison with literature we propose the following formation mechanism: chromium oxide (Cr_2_O_3_ or possibly CrO) formation may take place during attrition milling and SPS processes at the surface of steel grains. During the sintering process, chromium oxide phases react with the dispersed Al_2_O_3_ particles mixed with steel debris (1–2 micron particles resulted from milling). The spinel crystallites were surrounded by amorphous silica formed from Si which diffused from the steel alloy during the SPS process. This finding that the transformation occurs in presence of amorphous silica is consistent with literature describing both geological occurrence of chromite and phases with spinel structure and in annealed glass composites in the presence of silica phase. Also should be noted that conditions like local temperature, mechanical pressure and oxygen content at the grain boundaries of steel grains during the spark plasma sintering may deviate from the nominal experimental parameters due to formation of “melt pockets” during the SPS process. This local temperature increase may also contribute to the phase transformation at the relatively low process temperature of 900 °C.

## Data Availability

The datasets used and/or analysed during the current study available from the corresponding author on reasonable request.
